# Clinical Outcome of Endoscopic and Endoscopic-Assisted Microscopic Removal of Glomus Tympanicum: A Multicenter Retrospective Study

**DOI:** 10.3390/jcm14072388

**Published:** 2025-03-31

**Authors:** Waitsz Chang, Xiaoxin Chen, Mohamed Badr-El-Dine, Khalid Al Zaabi, Xinzhang Cai, Qi Wang, Nicolas Cornu, Romain Kania, Michael Chi Fai Tong

**Affiliations:** 1 Department of Otorhinolaryngology, Head and Neck Surgery, The Chinese University of Hong Kong, Hong Kong SAR 999077, China; tanachan@link.cuhk.edu.hk; 2 Institute of Human Communicative Research, The Chinese University of Hong Kong, Hong Kong SAR 999077, China; 3 Department of Surgery, ENT Division, Sultan Qaboos University Hospital, University Medical City, Muscat 123, Oman; mbeldine@yahoo.com (M.B.-E.-D.); khaboore21.ka@gmail.com (K.A.Z.); 4 Department of Otolaryngology, Faculty of Medicine, University of Alexandria, Alexandria 21561, Egypt; 5 Department of Otorhinolaryngology, Head and Neck Surgery, Xiangya Hospital, Central South University, Changsha 410008, China; zeiss93@csu.edu.cn (X.C.); wangqi2000811@163.com (Q.W.); 6 Key Laboratory of Otolaryngology Major Disease Research of Hunan Province, Changsha 410008, China; 7 National Clinical Research Centre for Geriatric Disorders, Department of Geriatrics, Xiangya Hospital, Central South University, Changsha 410008, China; 8 Department of Otorhinolaryngology-Head and Neck Surgery, Hôpital Paris-Saclay, 91400 Orsay, France; n.cornu@gh-nord-essonne.fr; 9 Department of Otorhinolaryngology, Head and Neck Surgery, Lariboisière Hospital, University of Paris Cité, 75010 Paris, France; romain.kania@gmail.com

**Keywords:** endoscopic ear surgery, glomus tympanicum, middle ear paraganglioma, transcanal, microscopic surgery

## Abstract

**Objective**: Comparing the clinical outcomes of glomus tympanicum (GT) resections by transcanal endoscopic ear surgery (TEES), microscopic- and endoscopic-assisted microscopic approaches. **Methods**: Adult patients conducting exclusive operations for GT within the tympanomastoid cavity were recruited in this retrospective cohort study at five international tertiary referral centers. GT resections were conducted by TEES, microscopic- and endoscopic-assisted microscopic approaches based on modified Fisch–Mattox classifications. Clinical characteristics and surgical outcomes were recorded and analyzed. **Results**: A total of 46 patients were included. A longer operative time was seen in more advanced GTs (A1: 106.73 ± 9.33 min, A2: 133.21 ± 13.47 min, B1: 176.88 ± 18.69 min, *p* = 0.005), while no significant differences were observed in the mean operative times among various surgical approaches. Preoperatively, 89.1% of patients experienced pulsatile tinnitus, and 56.5% exhibited conductive hearing loss. Postoperatively, only one patient continued to experience tinnitus (*p* < 0.001), and two patients had persistent hearing loss (*p* < 0.001). Higher disease grades correlated with poorer preoperative air-conduction thresholds (*p* = 0.015), while the differences in air-conduction thresholds before and after surgery did not demonstrate statistical significance across different tumor stages (*p* = 0.894) and surgical approaches (*p* = 0.257). The median follow-up period was 4 years, and only one recurrent case was found (2.2%, 1/46), which was treated by TEES and involved a B1 glomus tumor. **Conclusions**: Similar and excellent surgical outcomes were found among the TEES and microscope- and endoscopic-assisted microscopic approaches in early-stage GTs.

## 1. Introduction

Glomus tympanicum (GT), are benign tumors of neural crest cell origin arising from Jacobson’s nerve or Arnold’s nerve on the promontory [1]. They can extend to the mesotympanum, hypotympanum, mastoid, Eustachian tube, and external auditory canal but without involvement of the jugular bulb [2].

Patients diagnosed with glomus tumors typically report symptoms such as pulsatile tinnitus and/or hearing impairment, followed by otalgia and aural fullness [3]. Conductive hearing loss is often observed due to the tumor’s expansion into the mesotympanum, while the extent of labyrinthine invasion influences the severity of the sensorineural hearing loss and vertigo experienced by the patient [4]. Various classification systems for GTs have been suggested, with the most widely recognized being those put forth by Fisch and Mattox [5], as well as the system developed by Glasscock and Jackson [6]. The Fisch classification was subsequently refined by Mario Sanna, who provided a comprehensive characterization of tumors and their extent of involvement. This enhancement facilitates surgical planning and is referred to as the modified Fisch–Mattox classification [2]. The early-stage GTs classified by this modified classification was adopted in our study. Class A1 refers to tumor margins are clearly visible on otoscopic examination; Class A2 means tumor margins are not visible on otoscopy and may extend anteriorly to the Eustachian tube and/or to the posterior mesotympanum; Class B1 indicates tumors involving the middle ear with extension to the hypotympanum.

Surgery is often the first choice for GTs, even if radiotherapy and observation are alternatives for those who refuse surgery or are not suitable because of other diseases. Tumor resection can be performed via a transcanal, endaural, or post-auricular approach with the assistance of microscope, endoscope, exoscope, or a combination of these instruments [7]. The surgical approach for resection of GT is generally determined by the extension and location of the tumor and the preference of the surgeon [7]. The visualization modality of a microscope that allows for two-handed instrumentation is the traditional method used to surgically manage GT. The transcanal microscopic approach is typically used for smaller tumors (Glasscock–Jackson I and II and modified Fisch–Mattox A1 tumor stage), while larger tumors often require postauricular and occasionally bone removal, a considerable operating time and a longer hospital stay [2,6].

However, in some patients with early-stage tumors, the traditional microscopic transcanal approach may not be able to reach hidden regions such as the posterior epitympanum and the retrotympanum, so a microscopic transmastoid approach is needed [8]. Remarkably, in these cases, transcanal endoscopic ear surgery (TEES) may be a preferable option, providing good visualization for GTs and a panoramic view of the entire middle ear [9]. The endoscopic transcanal approach, an alternative, potentially less morbid method for addressing temporal bone pathology [10], is a newer technique in the treatment of GT limited to the middle ear cleft. Previous studies have proved it feasible and with low risk for patients with GTs of types A1, A2, and B1 (modified Fisch–Mattox classification) to undergo TEES [8,11,12]. However, hemostasis in the surgical field is more challenging to achieve during endoscopic procedures compared to microscopic surgical techniques [13,14].

The objective of this study is to conduct a comparative analysis of operative times, surgical outcomes, recurrence rates, and complications associated with GT resection by TEES and microscopic- and endoscopic-assisted microscopic approaches.

## 2. Materials and Methods

### 2.1. Patients’ Selection

A multicenter, retrospective cohort study was conducted at five international medical centers covering Asia, Africa, and Europe from 2014 to 2023. The informed consents for patients were waived in the retrospective study based on each medical center’s ethic policies.

The inclusion criteria were as follows: (a) patients were aged over 18 years old; (b) Patients had undergone exclusive operations for the tumor, which were pathologically confirmed to be glomus tympanicum; (c) patients with early-stage tumors (modified Fisch–Mattox classifications A1, A2, and B1) evidenced by high resolution computed tomography (HRCT) or magnetic resonance imaging (MRI).

The exclusion criteria were as follows: a. patients with type B2 and advanced GT; b. patients who failed to attend follow-up appointments, resulting in incomplete medical records.

### 2.2. Glomus Tympanicum Evaluation

**Figure 1:**
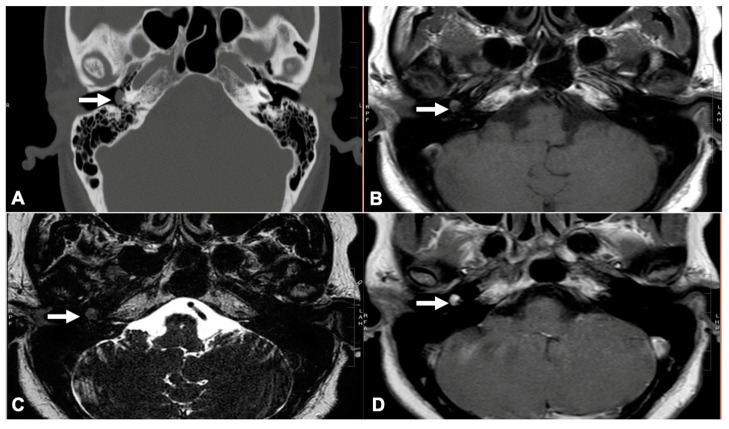
(**A**) A glomus tympanicum (white arrow) appears as a homogeneous soft tissue mass located at the cochlear promontory surface, as observed on the axial HRCT; (**B**) The glomus tympanicum (white arrow) in non-enhanced T1-weighted MRI; (**C**) The glomus tympanicum (white arrow) in T2-weighted MRI; (**D**) The glomus tympanicum (white arrow) limited in the middle ear cleft in contrast-enhanced T1-weighted MRI. HRCT, high-resolution computed tomography; MRI, magnetic resonance imaging.

We employed the modified Fisch–Mattox classification system. Preoperative otoscopic examinations indicated the presence of a red mass located behind the tympanic membrane (Supplementary Figure S1A). All patients underwent preoperative imaging, specifically contrast-enhanced HRCT or MRI, to identify GTs based on the detection of an enhancing soft tissue mass within the middle ear (Figure 1). This imaging was also utilized to assess the extent of the lesion and to rule out any involvement of the jugular bulb [15,16].

None of the patients underwent preoperative angiography and embolization. All patients had completed surgery for tumor resection, with the pathology confirming a paraganglioma diagnosis. All surgical procedures were conducted by at least one senior surgeon.

The following information was documented: (a) demographic (age, sex, and center); (b) tumor features (side, location, extension, and modified Fisch–Mattox classification); (c) patient characteristics (symptoms, tympanic membrane state, and facial nerve function according to House–Brackmann scale [17]); (d) hearing levels (preoperative and postoperative); (e) surgical characteristics (surgical approach and operative time); (f) surgical outcomes (completeness of resection, improvement in symptoms, facial nerve function, and complications and recurrence of tumor). Tumor location and extension were evaluated by radiological results and operation reports. Surgical approach, operative time, completeness of resection, chorda tympani state, and tympanic membrane status after repositioning the tympanomeatal flap were assessed according to the operative reports and videos. The air-conduction and bone-conduction pure tone average (PTA) was calculated by 500, 1000, 2000, and 4000 Hz. All patients were followed-up for at least 12 months, and the surgical outcome was recorded at the last follow-up.

The recurrence of GT was identified through clinical signs [18], such as the reappearance of symptoms and the observation of a reddish mass behind the eardrum during an otoscopic examination or through imaging results, which showed new or remaining enhancing lesions at the surgical site on CT or MRI scans [19].

### 2.3. Surgical Technique

The TEES were conducted by 3 mm in diameter, 14 cm in length, and 0-, 30-, and 45-degrees rigid endoscopes (Karl Storz, Tuttlingen, Germany). The microscope was prepared based on surgical plans. The canal skin was incised with a round canal knife from an 11 o’clock to 6 o’clock full thickness down to the bony meatus. The tympanomeatal flap was gently elevated from the bony meatus with an elevator dissector to the tympanic annulus. And the fibrous annulus was elevated to maximize exposure of the middle ear cavity, leaving only the anterior flap attached to the bony meatus. If the GT was filling the middle ear cleft, a 360-degree degloving of the tympanic membrane (TM) was performed [8]. The middle ear cavity was then visualized with a 0-degree endoscope to appreciate the extent of the tumor (Supplementary Figure S1). The bleeding was controlled by micro-bipolar and suction (Supplementary Figure S2A). After the tumor was removed, a final look would be performed to identify any residual tumor in hidden areas and the intactness of the ossicles (Supplementary Figure S2B,C). When a tympanic bony defect was found in the territory of the internal carotid artery, we used perichondrium to repair the bony defect (Supplementary Figure S2D,E). Ossicular chain repair was performed if the ossicle had to be removed (Supplementary Figure S2F). Eventually, the middle ear cavity was packed with gel foam and the tympanomeatal flap was repositioned. If a perforation of the TM was found at the end of the surgery, an endoscopic myringoplasty was performed at the same time using tragal cartilage perichondrium. The same TEES surgical technique described in this section was followed by the surgeons at all five centers.

### 2.4. Statistical Analysis

All results were performed using SPSS 28.0 (IBM, Chicago, IL, USA). Descriptive and frequent variables were used to describe patient demographics and characteristics. T-tests and one-way ANOVAs were used to compare the means between two or more groups. A chi-square test was utilized to compare the categorical variables among different groups. *p* < 0.05 was considered to be statistically significant.

## 3. Results

### 3.1. Patients’ Characteristics

A total of 46 patients from five international medical centers were included in the study. The median age of the participants was 52 years, with a predominance of female patients, accounting for 82.6% of the sample. All patients received confirmation of GT through pathological examinations. Notably, 54.3% of the tumors were situated on the left side. Utilizing the modified Fisch–Mattox classification, 15 patients were categorized as A1, 14 tumors as A2, and 17 patients as B1. The median time for follow-up was four years. The clinical characteristics of the patients are detailed in Table 1.

### 3.2. Relationship Between Disease Stage and Surgical Approach

**Figure 2:**
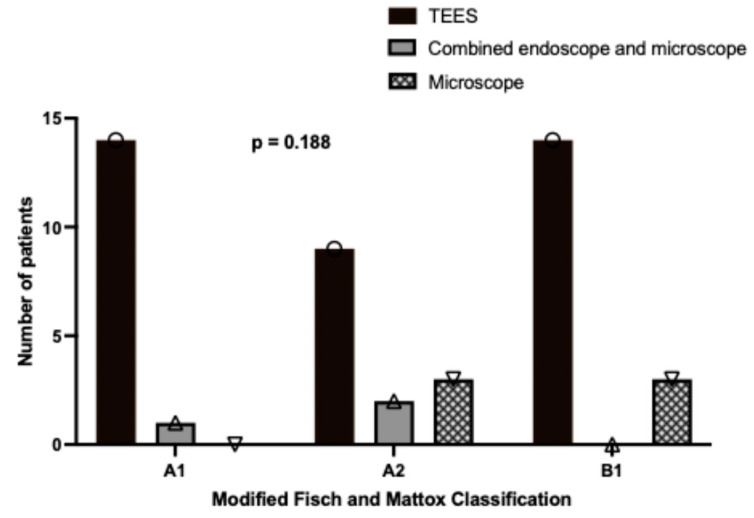
Relationship between the modified Fisch and Mattox classifications and the surgical approaches (N = 46). TEES, transcanal endoscopic ear surgery.

**Figure 3:**
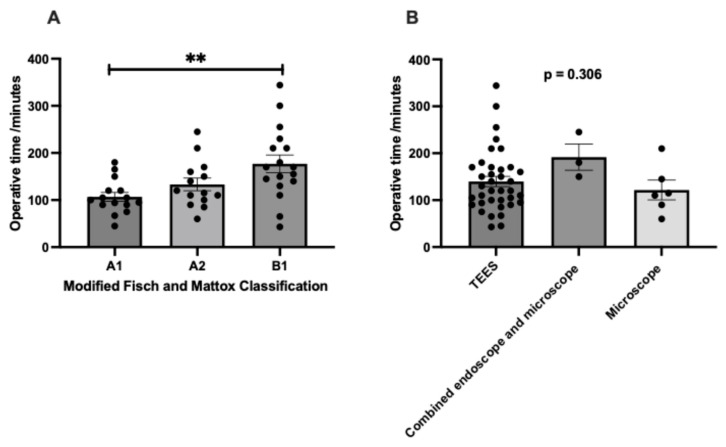
(**A**) Operative time of different modified Fisch and Mattox classifications; (**B**) operative time of different surgical approaches. TEES, transcanal endoscopic ear surgery. ** *p* < 0.01, N = 46.

As illustrated in Figure 2, a higher proportion of patients with A1 (93.3%, 14/15), A2 (64.3%, 9/14), and B1 (83.4%, 14/17) tumors underwent TEES. In contrast, the microscopic approach was used for a smaller percentage of cases involving more advanced tumors, specifically A2 (35.7%, 5/14) and B1 (17.6%, 3/17). However, these findings did not reach statistical significance. The average operative time for all patients was recorded as 140.72 ± 64.40 min (mean ± standard deviation). A one-way ANOVA analysis, as shown in Figure 3A, revealed significant differences in the mean operative times across various disease stages (mean ± standard error of the mean, as follows: A1: 106.73 ± 9.33 min, A2: 133.21 ± 13.47 min, B1: 176.88 ± 18.69 min, *p* = 0.005). Specifically, the Bonferroni post hoc test indicates that the surgical time for a B1 glomus was significantly longer than that for an A1 glomus (*p* = 0.004). Conversely, no significant differences were observed in operative times among the different surgical approaches, as depicted in Figure 3B (mean ± standard error of the mean, as follows: TEES: 139.68 ± 10.91 min, combined endoscope and microscope: 191.67 ± 28.04 min, microscope: 121.67 ± 21.08 min, *p* = 0.306).

### 3.3. Comparisons of Characteristics Between Preoperative and Postoperative

We can see in Supplementary Table S1 that, prior to surgery, 89.1% (41/46) of the patients exhibited pulsatile tinnitus, 56.5% (26/46) experienced conductive hearing loss, and 6.5% (3/46) reported bleeding. Postoperatively, only one patient continued to experience tinnitus (*p* < 0.001), and two patients retained hearing loss (*p* < 0.001). Furthermore, 93.5% (43/46) of the patients were asymptomatic (*p* < 0.001).

**Figure 4:**
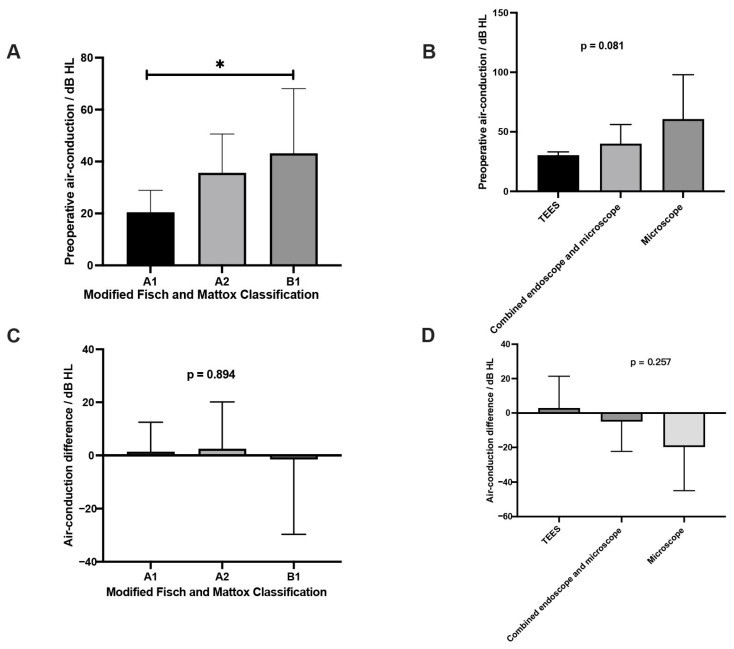
(**A**) The preoperative air-conduction threshold for the various modified Fisch and Mattox classifications; (**B**) the preoperative air-conduction threshold for the various surgical approaches; (**C**) the air-conduction threshold difference between the preoperative and postoperative state in different modified Fisch and Mattox classifications; (**D**) the air-conduction threshold difference between preoperative and postoperative states for the various surgical approaches. TEES, transcanal endoscopic ear surgery. * *p* < 0.05; N = 33.

The mean air-conduction threshold prior to surgery was recorded at 33.10 ± 19.46 dB HL, while the postoperative mean was 32.20 ± 25.22 dB HL. Statistical analysis revealed no significant difference between these two measurements (*p* = 0.818, Supplementary Table S1). Before the surgery, there was a discernible trend indicating that higher disease grades correlated with poorer air-conduction thresholds, as shown in Figure 4A (mean ± SEM, A1: 20.45 ± 2.54 dB HL, A2: 35.67 ± 4.51 dB HL, B1: 43.16 ± 7.52 dB HL, *p* = 0.015). While the preoperative air-conduction threshold was not associated with the surgical approaches, as seen in Figure 4B (mean ± SEM, TEES: 30.38 ± 2.80 dB HL; endoscopic-assisted microscope: 40.00 ± 16.07 dB HL; microscope: 60.70 ± 37.30 dB HL; *p* = 0.081). Furthermore, the differences in air-conduction thresholds before and after surgery did not demonstrate statistical significance across different tumor stages (Figure 4C, difference mean ± SEM, A1: 1.36 ± 3.35 dB HL, A2: 2.49 ± 5.31 dB HL, B1: −1.48 ± 8.50 dB HL, *p* = 0.894). When examining various surgical approaches, the differences in air-conduction thresholds from preoperative to postoperative assessments were also not statistically significant (Figure 4D, difference mean ± SEM, TEES: 2.88 ± 3.50 dB HL; endoscopic-assisted microscope: −5.00 ± 10.00 dB HL; microscope: −19.8 ± 17.8 dB HL; *p* = 0.257).

Preoperatively, all patients demonstrated normal facial nerve function. Postoperatively, only one patient experienced transient facial nerve palsy (House–Brackmann grade III), which showed partial improvement with corticosteroid treatment after three weeks. Notably, this patient was the only one for whom the surgeon employed low-power monopolar cautery to control bleeding from the tympanic sinus during TEES (Supplementary Table S1).

A total of 28 patients had their tympanic membrane state recorded during the operation. Tympanic membrane perforation was observed in three patients after repositioning to the tympanomeatal flap, representing a rate of 10.7% (3/28) during the intraoperative period. Myringoplasty utilizing perichondrium was performed on these three patients to address these perforations. Additionally, 14 patients with intact yet thin tympanic membranes underwent myringoplasty to reinforce the structural integrity of their membranes; this involved the use of cartilage–perichondrium grafts in six cases and perichondrium grafts in eight cases. Upon follow-up, three patients with intact but thin tympanic membranes who did not receive reinforcement during surgery subsequently developed perforations. Of these, one patient opted for myringoplasty, while the other two declined reconstructive surgery. Consequently, at the final follow-up assessment, two patients were identified as experiencing tympanic membrane perforations (Supplementary Table S2).

### 3.4. Recurrence of Paraganglioma

All patients underwent clinical or radiological follow-up for a minimum duration of 12 months. The overall recurrence rate of paraganglioma, following a median follow-up period of 4 years, was found to be 2.2% (1/46). The only one recurrent case was managed through TEES (Table 2) and involved a B1 GT (Table 3). The recurrence of the GT was identified one year postoperatively, presenting as a red mass located in the posterosuperior region above the stapes area. This mass was identified as a residual tumor that had been overlooked during the initial surgical intervention. The patient, a 73-year-old individual, chose to pursue observational follow-up rather than surgical intervention, despite being advised to consider a revision via a postauricular approach.

## 4. Discussions

GT is known to be the most common tumor of the middle ear, with slow growing, benign, and highly vascular characteristics [3]. Four to five times more females suffered from GTs compared to males in our study, similar to previous results [18,19]. The data utilized in this study were sourced from five international centers located in Asia, Europe, and Africa. Although the sample size at each center was relatively limited due to the numerous cases that were lost during the COVID-19 Pandemic, the findings nonetheless offer substantial evidence regarding the efficacy of both endoscopic and microscopic surgical approaches for GTs across diverse geographical regions. Additionally, two multicentric studies have been conducted regarding the endoscopic management of GTs [8,12]. However, both studies featured smaller sample sizes than our present investigation, with one study comprising 23 patients and the other 30 patients. Furthermore, these studies did not assess the differences in outcomes between the TEES and the microscopic approach. It was observed that a single-center study enrolled four times the number of patients compared to our investigation. This study assessed the typical presenting symptoms, clinical findings, imaging characteristics, outcomes of surgical management, and treatment-related complications for stage A to B GTs, while a comparative analysis of various surgical approaches was not conducted [19]. Therefore, we here to present the collective experiences of our collaborating members regarding the surgical management of early-stage GTs.

It is challenging to remove the GTs completely considering the complicated vascular nature and the important structures close to the tumor in the tympanic cavity [2]. While the radioresistant characteristics of these tumors, along with complications associated with radiation therapy, including stenosis of the external ear canal, radiation-induced neoplasms, and osteoradionecrosis, must be taken into account prior to administering radiation treatment to patients [20]. Surgical intervention is the first option for tumors limited in the tympanomastoid confines [19]. Radiosurgery appeared to be a good alternative or adjuvant to microsurgical resection in patients who are not amenable to complete surgical eradication [21].

Multiple approaches are available for the resection of GTs. The disease stage is the most important factor in choosing an approach, such as either transcanal or post-auricular, either using endoscope or microscope or both [19]. Traditionally, GTs restricted in the promontory were recommended to a microscopic transcanal approach. A postauricular approach with or without a hypotympanotomy or mastoidectomy was needed for tumors filling the whole mesotympanum. Tumors reaching the hypotympanum or extend into the mastoid cavity required a tympanomastoidectomy [2,9,22]. Tumors extending into the mastoid cavity and the external auditory canal were offered a canal wall down tympanomastoidectomy or closure of the external ear canal [9]. Ansley J. et al. compared the outcomes of microscopic and endoscopic transcanal GT resection involving 29 cases with a microscopic approach and 9 cases with an endoscopic approach [7]. Both approaches had high rates of gross tumor resection with similar hearing outcomes, operative times, and no recurrence. Fu et al. conducted a review of 18 cases of early-stage GTs treated with microscopic surgery and 7 cases treated with endoscopic surgery. Their findings indicate that there were no significant differences in the operative times, hospitalization costs, complications, or recurrence rates between the two surgical approaches [14]. Among numerous surgical approaches, the transcanal approach leads to the least morbidity. The microscope allows surgeon to operate using both hands, while the endoscope provides excellent visualization of the entire middle ear [23,24,25]. Previous studies have fully demonstrated the indications and advantages of resecting GTs of early stages by TEES. Marchioni et al. were the first to report the application of transcanal endoscopic approach for benign lesions that involve the middle ear cleft, such as GT tumors [25]. Fountarlis et al. presented three comprehensive cases involving the endoscopic management of GTs and concluded that endoscopic ear surgery is a safe and effective approach for the treatment of GTs. However, they identified tumor size as a significant limitation in this method [26]. A multicentric case series analyzed by Fermi et al. reported 30 patients with type A1, A2, and B1 GTs that underwent TEES, and the gross total resection reached 90% without recurrence of the tumor after a mean of 3 years for follow-up [8]. Ozgur et al. recruited 23 patients with stages 1 and 2 GTs (Glasscock–Jackson classification) undergoing the TEES and found that TEES was linked to short operative durations, low risk of complications, and rapid discharge [12].

In our multicentric study, we compared the clinical characteristics and surgical outcomes of early-stage GT resections by TEES and microscopic- and endoscopic-assisted microscopic approaches. The disease stage was defined by modified Fisch–Mattox classification. A total of 38 patients were subjected to transcanal surgical techniques, which comprised 37 TEES involving 14 A1 tumors, 9 A2 tumors, and 14 B1 tumors, as well as one endoscopic-assisted microscopic approach for an A2 tumor. Additionally, eight patients received a post-auricular approach, which included two endoscopic-assisted microscopic procedures (one for an A1 tumor and one for an A2 tumor) and six microscopic approaches (three for A2 tumors and three for B1 tumors). The selection of the surgical approach for each patient was based on the extent of the tumor and the preferences of the surgeon. Besides, surgeons from medical centers in Egypt and Oman have routinely reinforced the tympanic membrane at the end of surgical procedures. This practice is implemented to mitigate the elevated incidence of tympanic membrane perforation postoperatively, particularly when the tympanic membrane is found to be intact yet thin following the repositioning of the tympanomeatal flap at the end of the surgery.

Referring to the clinical symptoms caused by the tumor, pulsatile tinnitus and conductive hearing loss occurred most frequent, and most of these symptoms were resolved after surgery in our study. Only one patient continued to experience pulsatile tinnitus, and two patients retained conductive hearing loss, one of whom, a recurrent case, experienced persistent pulsatile tinnitus and hearing loss. There was a red mass seen on the posterosuperior region above the stapes area, which was a residual tumor missed from the first surgery. Another case retained hearing loss due to the inability of the ossicles to be conserved during the surgery. As for the hearing evaluation, no significant air-conduction threshold differences were found between preoperative and postoperative states within different surgical approaches and disease stages. However, we noticed the preoperative air-conduction threshold was determined by the different disease stages. The more advanced the disease stage, the higher the air-conduction threshold. This is in consistent with the findings of previous study [12].

The operative time was found to be affected by the stage of the disease, while no correlation was observed with the surgical techniques employed in our study. Specifically, more advanced stages of the disease were correlated with prolonged operative times. The complexity of the advanced disease, coupled with the constraints of the limited working space, contributed to the challenges encountered during the surgical process, thereby significantly extending the operative time. These results are consistent with findings from other research studies [7,14,27]. Furthermore, the complications observed, such as facial nerve palsy and tumor recurrence, were not linked to the surgical techniques employed or the early stages of the tumor, with one patient having transient HB grade-3 facial nerve palsy after the surgery. This was attributed to the use of low power monopolar cautery used to stop deep seated bleeding near the tympanic sinus. Even with low power, the monopolar would generate higher cautery than bipolar, which may cause nerve damage [28]. Since then, monopolar was never used for hemostasis, and none of the left patients had facial nerve functional problems. Therefore, it is essential to regulate the cautery of instruments during tumor resection or hemostasis in order to prevent injury to the facial nerve or cochlea [19]. Most surgeons use bipolar electrocautery for the safe excision of paraganglioma tumors from the middle ear, and different types of lasers, including CO2 lasers [29], diode lasers [30], KTP lasers [31], and Nd-YAG lasers [32], as well as new technique like coblation [33], have been used for excision to avoid bleeding. Additionally, only one patient experienced tumor recurrence at 1-year follow-up due to a residual tumor missed in the first surgery. The complete surgical removal of early-stage tumors should be given significant consideration.

Finally, the selection of either the TEES or microscopic approach did not significantly affect operative times, hearing outcomes, symptomatology, or complications associated with early-stage GTs. However, postauricular excision in microscopic surgery may influence the aesthetic appearance and recovery of the wound, whereas endoscopic surgery may present challenges in achieving hemostasis and conducting the procedure effectively in instances of substantial hemorrhage or a constricted ear auditory canal. Therefore, a thorough preoperative assessment and the capacity to transition between endoscopic and microscopic techniques as necessary are essential for optimizing patient outcomes.

Nevertheless, our study has several limitations. Primarily, the research was conducted as a retrospective analysis with a limited sample size within each subgroup. Consequently, the statistical evaluation of the various surgical techniques and disease stages was inevitably confounded by the small number of participants in each subgroup. To validate the recurrence rates associated with different surgical approaches, further large-scale and longitudinal studies are warranted.

## 5. Conclusions

Similar clinical characteristics and surgical outcomes were found among the TEES, microscopic approach, and endoscopic-assisted microscopic approach in early-stage GTs. The advanced stages of the tumors were the main factors contributing to the long operative times and poor hearing levels.

## Figures and Tables

**Table 1:** Patients’ characteristics.

Characteristic	N = 46, %/Median (Range)
Age (years)	52 (22–83)
Gender	
Female	82.6% (38/46)
Male	17.4% (8/46)
Side	
Left	54.3% (25/46)
Right	45.7% (21/46)
Center	
Hong Kong, China	17.4% (8/46)
Mainland, China	30.4% (14/46)
Paris, France	10.9% (5/46)
Egypt	37.0% (17/46)
Oman	4.3% (2/46)
Stage (Modified Fisch and Mattox Classification)	
A1	32.6% (15/46)
A2	30.4% (14/46)
B1	37.0% (17/46)
Surgical approach	
TEES	80.4% (37/46)
Endoscopic-assisted microscope	6.5% (3/46)
Microscope	13.0% (6/46)
Follow-up time (years)	4 (1–11)

**Table 2:** Recurrence rates for the various surgical approaches (N = 46).

Surgical Approach	Recurrence Rate	*p*-Value
TEES	2.7% (1/37)	>0.05 ^ab, ac^
Endoscopic-assisted microscope	0% (0/3)	>0.05 ^ab, bc^
Microscope	0% (0/6)	>0.05 ^ac, bc^

Each superscript letter denotes a subset of surgical approaches: a means TEES, b means endoscopic-assisted microscopic approach, and c means microscope. TEES, transcanal endoscopic ear surgery.

**Table 3:** Recurrence rates in different modified Fisch and Mattox classifications (N = 46).

Stage	Recurrence Rate	*p*-Value
A1	0% (0/15)	>0.05 ^ab, ac^
A2	0% (0/14)	>0.05 ^ab, bc^
B1	5.9% (1/17)	>0.05 ^ac, bc^

Each superscript letter denotes a subset of modified Fisch and Mattox classifications: a means A1, b means A2, c means B1.

## Data Availability

The data presented in this study are available on request from the corresponding author due to privacy.

## Supplementary Materials

The following supporting information can be downloaded at: https://www.mdpi.com/article/10.3390/jcm14072388/s1. Table S1: Comparison of patients’ hearing, facial nerve function and tympanic membrane state between preoperative and postoperative. Table S2: Comparison of patients’ symptoms between preoperative and postoperative (N = 46). Figure S1: Left preoperative otoscopic graph showed a red protympanic mass. (B) A transcanal endoscopic view of the A2 glomus tympanicum in protympanum and mesotympanum. (C) Total resection of the tumor from the tympanic cavity. ty, tympanic membrane; gl, glomus tympanicum; in, incus; ma, malleus. Figure S2: (A) A transcanal endoscopic view of stopping glomus tympanicum bleeding using bipolar forceps and suction. (B) The glomus tympanicum located in the epitympanum and extended to the eustachian tube. (C) Removing the incus to completely clearing the tumor from tympanic cavity. (D) Tympanic bony defect was found in the territory of the internal carotid artery. (E) A perichondrium was used to repair the bony defect. (F) A cartilage was grafted to compose of ossicle. Green circle means suction, and black triangle means micro-bipolar. gl, glomus tympanicum; in, incus; ma, malleus; in, incus; st, stapes; ca, carotid artery; fn, facial nerve; pr, promontory; ttc, tensor tympani canal; gr, graft cartilage.
