# Supplementary material for: Clinical Outcome of Endoscopic and Endoscopic-Assisted Microscopic Removal of Glomus Tympanicum: A Multicenter Retrospective Study
Source: J Clin Med. 2025 Mar 31;14(7):2388. doi: 10.3390/jcm14072388 (PMC11989738; doi:10.3390/jcm14072388)
Supplement: Supplementary file 1 [file jcm-14-02388-s001.zip › jcm-3520926-supplementary.pdf]

Supplementary Table S1 Comparison of patients' hearing, facial nerve function and tympanic membrane state between preoperative and postoperative.

	Preoperative	Postoperative	P value
Air-conduction threshold (dB HL)	33.10 ± 19.46	32.20 ± 25.22	0.818
House-Brackmann facial nerve grading (%)			1.000
I	100% (46/46)	97.8% (45/46)	-
II	0% (0/46)	0% (0/46)	-
III	0% (0/46)	2.2% (1/46)	-
IV	0% (0/46)	0% (0/46)	-
V	0% (0/46)	0% (0/46)	-
VI	0% (0/46)	0% (0/46)	-
Tympanic membrane state (perforation %)	0% (0/46)	4.3% (2/46)	0.495

Supplementary Table S2 Comparison of patients' symptoms between preoperative and postoperative (N = 46).

Symptoms	Preoperative	Postoperative	P value
Pulsatile tinnitus	89.1% (41/46)	2.2% (1/46)	< 0.001*
Hearing loss	56.5% (26/46)	4.3% (2/46)	< 0.001*
Bleeding	6.5% (3/46)	0% (0/46)	0.242
Aural fullness	0% (0/46)	0% (0/46)	-
Vertigo/dizziness	2.2% (1/46)	0% (0/46)	1.000
Other (itchy and otalgia)	2.2% (1/46)	0% (0/46)	1.000
Asymptomatic	2.2% (1/46)	93.5% (43/46)	< 0.001*

*, $p < 0.05$.

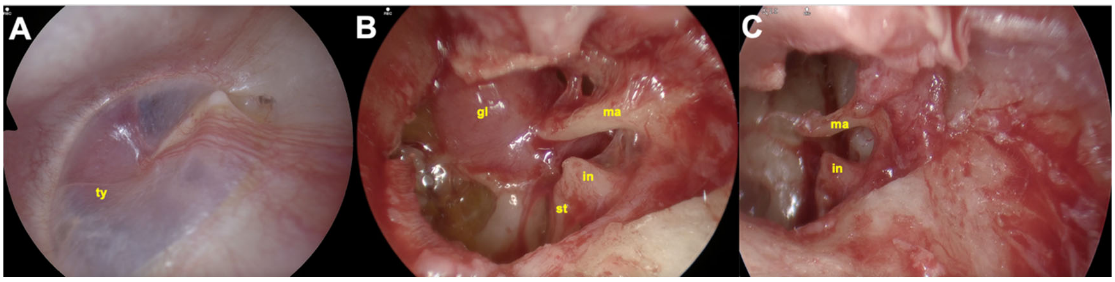

Supplementary Figure 1 (A) Left preoperative otoscopic graph showed a red protympanic mass. **(B)** A transcanal endoscopic view of the A2 glomus tympanicum in protympanum and mesotympanum. **(C)** Total resection of the tumor from the tympanic cavity. ty, tympanic membrane; gl, glomus tympanicum; in, incus; ma, malleus.

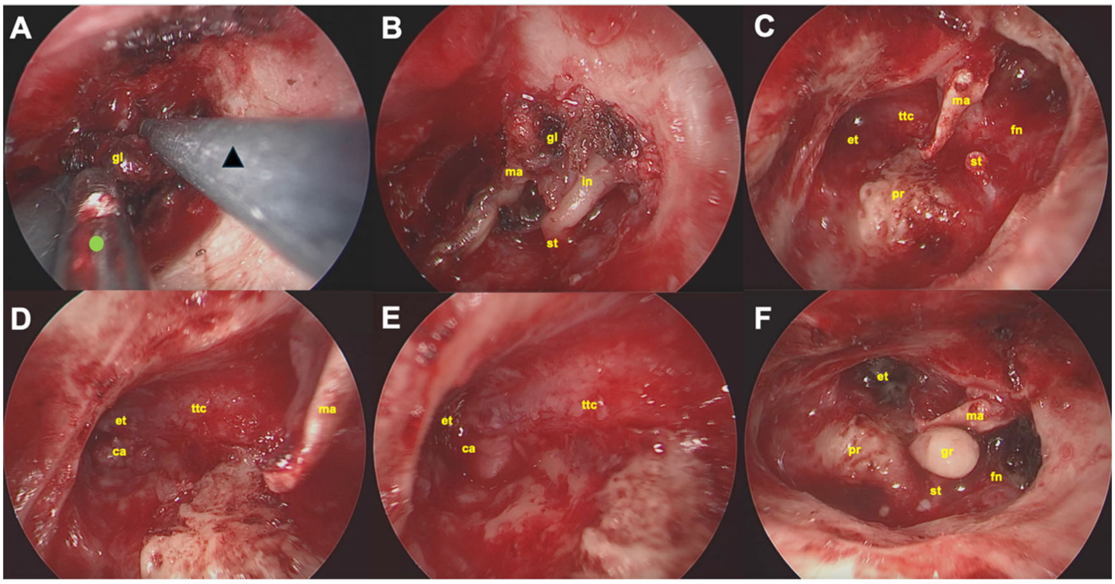

Supplementary Figure 2 (A) A transcanal endoscopic view of stopping glomus tympanicum bleeding using bipolar forceps and suction. **(B)** The glomus tympanicum located in the epitympanum and extended to the eustachian tube. **(C)** Removing the incus to completely clearing the tumor from tympanic cavity. **(D)** Tympanic bony defect was found in the territory of the internal carotid artery. **(E)** A perichondrium was used to repair the bony defect. **(F)** A cartilage was grafted to compose of ossicle. Green circle means suction, and black triangle means micro-bipolar. gl, glomus tympanicum; in, incus; ma, malleus; in, incus; st, stapes; ca, carotid artery; fn, facial nerve; pr, promontory; ttc, tensor tympani canal; gr, graft cartilage.
